# Structural and parameter uncertainty in Bayesian cost-effectiveness models

**DOI:** 10.1111/j.1467-9876.2009.00684.x

**Published:** 2010-03

**Authors:** Christopher H Jackson, Linda D Sharples, Simon G Thompson

**Affiliations:** Medical Research Council Biostatistics UnitCambridge, UK

**Keywords:** Bayesian model comparison, Health economics, Markov chain Monte Carlo methods, Model averaging, Model uncertainty

## Abstract

Health economic decision models are subject to various forms of uncertainty, including uncertainty about the parameters of the model and about the model structure. These uncertainties can be handled within a Bayesian framework, which also allows evidence from previous studies to be combined with the data. As an example, we consider a Markov model for assessing the cost-effectiveness of implantable cardioverter defibrillators. Using Markov chain Monte Carlo posterior simulation, uncertainty about the parameters of the model is formally incorporated in the estimates of expected cost and effectiveness. We extend these methods to include uncertainty about the choice between plausible model structures. This is accounted for by averaging the posterior distributions from the competing models using weights that are derived from the pseudo-marginal-likelihood and the deviance information criterion, which are measures of expected predictive utility. We also show how these cost-effectiveness calculations can be performed efficiently in the widely used software WinBUGS.

## 1. Uncertainty in health economic decision models

Cost-effectiveness models are now routinely used by health policy makers to evaluate medical interventions and to allocate resources. These are often discrete time Markov models for the occurrence of clinical events (Briggs *et al.*, 2006). Each event or clinical state is associated with a monetary cost and a measure of benefit such as quality-adjusted life. The model parameters are usually informed by several sources of data, including clinical trials, hospital registers and population mortality statistics. The aim of the model is to estimate the long-term expected cost and benefit of one treatment compared with another, which are a complex function of the model parameters. This result is subject to a range of uncertainties, including uncertainties about the choice of model and about the parameters of a chosen model. In these models, short-term evidence, typically from randomized trials with follow-up of a few years, must be extrapolated to many years or even patients’ lifetimes. A set of alternative models which are all plausible in the short term may produce widely varying lifetime results.

Uncertainty about the parameters is normally incorporated by using probabilistic sensitivity analysis (Claxton *et al.*, 2002). However, uncertainty about the model structure itself is usually investigated by presenting the results under a series of alternative assumptions, but with no formal indication of their relative plausibility. Typical assumptions that are considered as ‘structural’ include the choice of clinical events or states and permitted transitions between them, the way in which each transition probability depends on patients’ characteristics or varies through time, or the choice of data that are used to inform the model (Bojke *et al.*, 2006). Where data exist to test each structural assumption, statistical methods for model assessment can be used to estimate the weight of evidence for each model structure, to aid the decision maker in their interpretation of the results. Jackson *et al.* (2009) discussed the use of model averaging to account for structural uncertainty in health economic models. Models were fitted by using maximum likelihood and their results weighted by using likelihood-based information criteria. In this paper, these methods for model uncertainty are generalized to a fully Bayesian setting. Bayesian models, in particular implemented by Markov chain Monte Carlo (MCMC) posterior sampling, are increasingly used in health policy evaluations, because of the convenient framework that they provide for synthesizing multiple sources of uncertain evidence (Spiegelhalter *et al.*, 2004).

We present a Bayesian model for the cost-effectiveness of two strategies for the prevention of cardiac arrhythmia and formally account for both parameter and model uncertainty. Firstly, the expected cost and benefit are complex functions of the model parameters. Normally in health economic models (Briggs *et al.*, 2006) probabilistic sensitivity analysis involves choosing distributions from standard families, often independent, to approximate the joint uncertainty about all model parameters. The resulting distribution of expected costs and benefits is accumulated via repeated sampling from the parameter distributions. Instead, by using fully Bayesian, one-stage model fitting and cost-effectiveness prediction, we can automatically sample from the required posterior distribution instead of approximating it. Parameter uncertainty is propagated formally to the model outputs, accounting for all correlations between the parameters. This method was previously demonstrated for simple Markov health economic models by Spiegelhalter and Best (2003) and Cooper *et al.* (2004), using the freely available software WinBUGS (Lunn *et al.*, 2000). One disadvantage of this implementation, which was cited by Cooper *et al.* (2004), is that it is computationally slow. We show how it can be adapted to handle arbitrarily complex model structures at little extra computational cost, by using an extension of WinBUGS (Lunn, 2003) that enables complex functions of parameters to be calculated substantially faster.

Secondly, this application involves several choices between plausible model structures, including the choice of covariates for the incidence of clinical events, and different parametric forms for the relationship of mortality to age. Rather than just presenting the results under each of these alternative scenarios, we take formal statistical account of model structure uncertainty. We assess the relative plausibility of these scenarios against data by estimating the expected predictive utility of each model by using the cross-validatory ‘pseudo-marginal-likelihood’ (PML) (Geisser and Eddy, 1979; Gelfand and Dey, 1994) and the commonly used deviance information criterion (Spiegelhalter *et al.*, 2002). The differences in these measures between models can be ‘calibrated’ by a Bayesian bootstrap procedure, to produce the probability that each model has the highest expected predictive utility for a replicate data set, among the models being compared. These probabilities can then be used to produce a model-averaged posterior distribution which allows for sampling uncertainty about model selection. This differs from the usual Bayesian model averaging procedure, in that we do not consider prior or posterior probabilities of the models being true, since we believe that the true process underlying our clinical history data is extremely complex.

In the following section, we introduce the application to cardiac arrhythmia, describe how cost-effectiveness is estimated and present a set of reasonable alternative models for the data. In Section 3 we describe how the posterior distributions of cost and effectiveness can be estimated directly, accounting for the uncertainty in the model parameters, and how this estimation can be performed efficiently in WinBUGS. In Section 4, we describe measures of Bayesian model adequacy that are based on expected predictive utility, which may be used to estimate selection probabilities for the alternative models and produce a model-averaged posterior distribution. The inferences from the individual models, the model adequacy measures and the model-averaged inferences for our application are presented in Section 5. We conclude with a discussion of further complexities of health economic decision models which may be addressed in this Bayesian framework.

The data that are analysed in the paper and the programs that were used to analyse them can be obtained from

## 2. Application: implantable cardioverter defibrillators

A Markov decision model has been developed to estimate the cost and effectiveness of implantable cardioverter defibrillators (ICDs), relative to anti-arrhythmic drug (AAD) treatment, for the prevention of cardiac arrhythmia among patients at high risk of sudden cardiac death (Buxton *et al.*, 2006). To inform this model, individual data from 535 patients implanted with ICDs at two UK centres were combined with individual data from 430 patients from the Canadian Implantable Defibrillator Study (O'Brien *et al.*, 2001) randomized controlled trial, of ICD implantation compared with treatment with the AAD amiodarone. The data consist of baseline demographic and risk factor information, longitudinal histories of hospital admissions and dates of death or censoring. The mean follow-up time was 3.68 years, with a maximum follow-up of 7.04 years, after ICD implantation or initiation of AAD treatment.

## 2.1. Base case Markov model

**Fig. 1:**
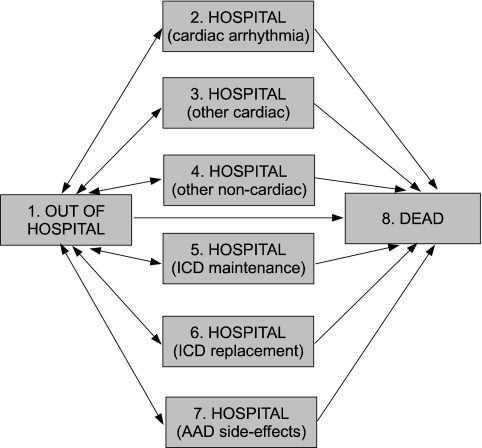
Daily Markov model for hospitalization and death among patients at risk of cardiac arrythmia: on the next day, patients can either remain in the same state or move to one of the permitted states indicated

The ‘base case’ model, which will be compared against alternative model structures, is an eight-state Markov model with a daily discrete time unit. This is described in detail elsewhere (Buxton *et al.*, 2006); a summary is presented here. The permitted transitions between the eight states are illustrated in Fig. 1. The states correspond to alive and out of hospital (state *r*=1), admission to hospital for six alternative reasons (states *r*=2,…,7) and death from any cause (state *r*=8). Several covariates are assumed to affect probabilities of hospital admission and mortality, via multinomial logistic regression. Let (*X*_*ijrs*_:*s*=1,…,8) be the number of observed transitions from state *r* to each state *s*, for patient *i* in year *j* after start of treatment, and 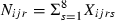. This is modelled as 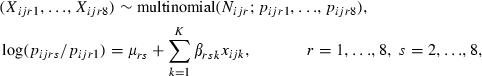
(1) where *p*_*ijrs*_ is the transition probability from state *r* to state *s*, for patient *i* in year *j* with covariates *x*_*ij*1_,…,*x*_*ijK*_. *p*_*ijrs*_=0 for transitions that are not indicated in Fig. 1, such as transitions from one hospital admission state to another, and *p*_*ijr*1_ is defined so that 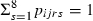. The transitions are aggregated by year *j* so that time-dependent covariates can be included, which are assumed to be constant within each year.

Four baseline binary covariates were considered: treatment (*β*_*rs*1_ is the effect of AAD treatment, compared with ICD), sex (*β*_*rs*2_ is the effect of female, compared with male), left ventricular ejection fraction LVEF (*β*_*rs*3_ is the effect of LVEF < 35%, compared with LVEF ≥35%) and country of treatment (*β*_*rs*4_ is the effect of treatment in the UK, compared with Canada). Age is treated as a time-dependent covariate with a piecewise constant effect (baseline: under age 60 years, *β*_*rs*5_ for age 60–70 years and *β*_*rs*6_ for age 70 years or over). Certain constraints were imposed on the covariate effects owing to the sparsity of data (Buxton *et al.*, 2006).

- Only two out of the 174 observed deaths followed a day spent in hospital; therefore the baseline risk of death and all effects on that risk were assumed to be independent of the state on the day before death, so *μ*_*r*8_=*μ*_D_ for all *r* and *β*_*r*8*k*_=*β*_D*k*_ for all *r* and *k*.
- The only covariate that is assumed to affect the length of stay in hospital is country of treatment, so *β*_*rrk*_=0 for all *k* other than *k*=4.
- No effect of country of treatment is assumed for the rate of hospital admission and the length of hospital stay for drug side-effects (state 7), so *β*_174_=*β*_774_=0. There were no admissions for this cause in the UK data.

Thus, in this base case model, there are 59 unknown parameters: *μ*_1*s*_,*s*=2,…,7(6); *β*_1*sk*_,*s*=2,…,6,*k*=1,…,6 (30); *β*_17*k*_,*k*=1,2,3,5,6 (5); *μ*_*rr*_,*r*=2,…,7 (6); *β*_*rr*4_,*r*=2,…,6 (5); *μ*_D_ (1); *β*_D*k*_,*k*=1,…,6 (6).

## 2.2. Prior distributions and implementation

The prior distribution for *β*_D1_, the treatment effect on mortality, was normal, mean 0.414, standard deviation 0.16, taken from a fixed effect meta-analysis of two previous randomized trials of ICD *versus* AAD treatment (AVID Investigators, 1997; Kuck *et al.*, 2000). Widely dispersed prior distributions (normal, mean 0, standard deviation 10) are assumed for all other parameters. The posterior distributions are estimated by using Gibbs and Metropolis MCMC sampling, in the WinBUGS software (Lunn *et al.*, 2000). The code that was required to run this base case model is available from http://www.blackwellpublishing.com/rss.

## 2.3. Cost-effectiveness estimation

The expected costs and benefits of ICD compared with AAD treatment for a ‘typical patient’ are estimated, as part of the model, as follows. The patient is defined by a set of covariate values, including age at ICD implant or initiation of AAD therapy. Let *P*_*t*_ be the transition probability matrix of the Markov model at day *t* for this patient, which is time dependent since it varies according to the patient's age, among other patient characteristics. The vector of probabilities *π*_*t*_ that a patient is in each state on day *t* follows the recursive relationship *π*_*t*_=*π*_*t*−1_*P*_*t*_, where *π*_0_ is such that an individual is in state 1 (out of hospital) with probability 1 at day 0. There is a vector of costs **c**_*u*_=(*c*_1*u*_,…,*c*_8*u*_) associated with 1 day spent in each of the eight states, and a fixed initial cost *c*_0*u*_, for ICD implantation (*u*=1) or drugs (*u*=0). Future costs are discounted (Briggs *et al.*, 2006) at a rate of 100*δ*% per day. Then the total expected cost over *T* days for treatment *u* is 
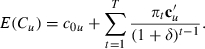
(2)

There is an analogous formula for the total expected benefit *E*(*B*_*u*_). In this example, benefit is expressed in quality-adjusted life years (QALYs), and the discount rate for both costs and QALYs was 3.5% per year. Life years were quality adjusted by using standard age-dependent utilities of 0.75 for all hospital and out-of-hospital states and both treatments. The daily costs that were used in the analysis, determined from the average experience of UK patients, are listed in Table 1.

**Table 1:** Costs for each state†

*State*	*Cost (£) for ICD (u = 1)*	*Cost (£) for AAD (u = 0)*
*c*_0*u*_: initial costs	23841	1566
*Daily costs*		
1, out of hospital	2.30	2.43
2, arrhythmic	526	
3, other cardiac	519.50	
4, non-cardiac	296.50	
5, ICD maintenance	646	
6, ICD replacement	5495.50	
7, AAD side-effects	122	
8, death	0	

†Costs for states 2–8 are common for ICD and AAD treatment.

The model is run twice, once assuming a policy of ICD implantation, and once with a policy of AAD therapy. The model is run for an ICD or AAD patient of average age, taken to be 63 years of age and with low LVEF. Around 87% of such individuals are male, so the binary ‘female’ covariate for the ‘typical’ patient is set to 0.13. The time horizon *T* is a ‘lifetime’ of 100 years after the starting age of 63 years. The targets of estimation are the incremental cost Δ_C_=*E*(*C*_1_)−*E*(*C*_0_) of ICD compared with AAD, the incremental benefit Δ_B_=*E*(*B*_1_)−*E*(*B*_0_), and the incremental cost-effectiveness ratio (ICER) Δ_C_/Δ_B_, interpreted as the additional cost per QALY of ICD compared with AAD.

## 2.4. Structural uncertainties

The Markov model that was described in Section 2.1 involves several uncertain choices of model structure. We fit several plausible variants of the base case model to examine the uncertainty surrounding the structural assumptions. The base case model is labelled *M*_1_, and the alternative models *M*_2_,…,*M*_10_.

## 2.4.1. Covariate selections

Covariate selection is a common form of model uncertainty. The base case model includes the effects of four time constant covariates and one time-dependent covariate on 19 possible transitions, with constraints which give a total of 59 unknown parameters. Including unnecessary covariate effects may lead to a model with high predictive mean-square error, and omitting necessary covariates may result in bias. Therefore, a set of three plausible alternative restrictions *M*_2_–*M*_4_ on the covariates was chosen, guided by the estimated covariate effects under the base case model. As each MCMC model fit took several hours, it would have been impractical to fit and compare models with every possible combination of covariates. There are alternative methods of constructing a single MCMC sampler which mixes over the space of competing models (see Section 6.5), but these require specialized programming.

- *M*_2_: an extra piecewise constant effect of age on length of stay in hospital is included, alongside the effect of country (12 parameters more than the base case).
- *M*_3_: all covariate effects on hospital admission for AAD side-effects, other than treatment, are removed (giving four fewer parameters than the base case).
- *M*_4_: all effects of sex are excluded, other than the effects on death and hospital admission for cardiac arrhythmia (five fewer parameters than the base case).

## 2.4.2. Extrapolation of age effects on survival

A particularly questionable assumption in the base case analysis is the piecewise constant dependence of mortality on age, with effects for age under 60, age 60–70 and age 70 years or over. The risk of mortality was assumed to be constant for all patients over 70 years old. In this population, the probability of survival beyond age 80 years (from Kaplan–Meier estimates) is about 20%; therefore different models for this age dependence may have a substantial effect on estimates of the treatment effectiveness in terms of quality-adjusted survival.

**Fig. 2:**
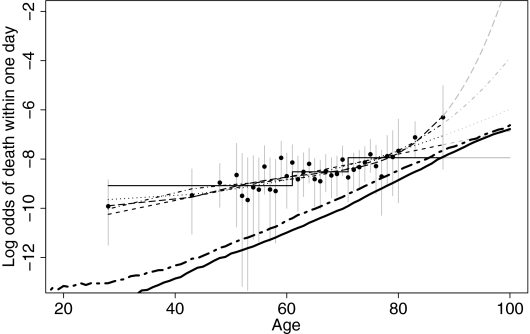
Unadjusted log-odds of death in 1 day (with 95% confidence intervals) against age for UK and Canada ICD study patients, calculated from counts of deaths per year (least-squares-fitted functions of log-odds of death as a function of age are illustrated, along with the log-odds of death for the general UK population; extrapolations beyond the range of the data are drawn in light grey): 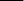, UK population (female); **-** **—** **-**, UK population (male); 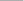, step function on three age periods; - - - - - -, linear; ……., quadratic; - · - · - · -, cubic; – – –, quartic

In Fig. 2, point estimates and confidence intervals for the log-odds of death within 1 day, calculated from yearly counts of deaths in the study population, are plotted against age. Only sparse data are available to inform plausible age–mortality models beyond age 80 years. Fig. 2 illustrates a series of polynomial regressions fitted to the yearly point estimates of log-odds. These all seem to fit the study data reasonably well, but there are potentially important differences between the models in the extrapolated log-odds among the oldest ages. Therefore, three alternative models are fitted in which the piecewise constant effect of age on each log-odds of transition (equation (1)) is replaced by polynomial functions:

- *M*_5_, a quadratic function of age (same number of parameters as the base case);
- *M*_6_, a cubic function of age (seven more parameters than the base case);
- *M*_7_, a quartic function of age (14 more parameters than the base case).

A further three models *M*_8_,*M*_9_ and *M*_10_ are fitted as extensions of *M*_5_,*M*_6_ and *M*_7_ respectively, which also include an interaction between all age terms and treatment. These have 14, 28 and 42 more parameters respectively than the base case.

## 3. Probabilistic sensitivity analysis

The effect of parameter uncertainty on the expected cost *E*(*C*) and expected benefit *E*(*B*) is accounted for by probabilistic sensitivity analysis. In cost-effectiveness models, this usually involves choosing distributions for all unknown parameters after the models have been estimated (Briggs *et al.* (2006), chapter 4). Monte Carlo simulation from these distributions then leads to distributions for *E*(*C*) and *E*(*B*). This is termed a *two-stage* method (Spiegelhalter and Best, 2003). Typically, standard distributions are chosen to approximate the marginal posterior of each parameter, such as beta distributions for probabilities and normal distributions for unbounded parameters, with means and variances inferred from data. Correlations between parameters are difficult to specify in practice and are often ignored.

In a fully Bayesian framework, however, the appropriate joint distribution can be automatically identified from the data. After convergence, MCMC simulation produces a sample from the joint posterior distribution of all parameters and functions of parameters, including *E*(*C*) and *E*(*B*). Thus model fitting and probabilistic sensitivity analysis can be accomplished in a *one-stage* method (which was termed a *comprehensive decision model* by Cooper *et al.* (2004)), avoiding the need to specify potentially inaccurate distributions for the parameters and accounting fully for parameter correlations.

The estimated distributions of *E*(*C*) and *E*(*B*) lead to a posterior *probability of cost-effectiveness* PCE(*λ*), which is defined as the probability of a positive incremental net benefit: 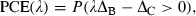
(3) where the ‘threshold’*λ* is the amount of money that a policy maker is willing to pay for 1 unit of benefit (such as a QALY).

## 3.1. Implementation in WinBUGS

The one-stage method for cost-effectiveness estimation was previously described by Spiegelhalter and Best (2003), who demonstrated how a simple example can be implemented in the WinBUGS software for MCMC sampling. In WinBUGS, users can define arbitrarily general models by using the BUGS language (Lunn *et al.*, 2000) and estimate the posterior distributions of functions of the model parameters. However, as noted by Cooper *et al.* (2004), this can be slow for complex cost-effectiveness models. For example, our analysis requires calculating the expected lifetime cost (equation (2)), which involves over 365 × 100 multiplications of 8 × 8 matrices, one for each daily time unit over 100 years. This is currently computationally infeasible when specified by using the BUGS language.

To perform this computation we use the WBDev interface (Lunn, 2003) which enables users to write extensions to the core WinBUGS software. This can be used to calculate complex deterministic functions of parameters, or to enable arbitrary univariate statistical distributions to be employed in models. By ‘hard-wiring’ these calculations into the software as compiled code, large computational savings can be made. This approach allows the benefits of Bayesian modelling, such as one-stage estimation and incorporating the results of previous studies, to be combined with arbitrarily complex cost-effectiveness calculations, which would have previously required laborious MCMC programming or approximations. The WBDev code for the base case model is available from http://www.blackwellpublishing.com/rss.

Alternatively, the exact posterior distribution of *E*(*C*) and *E*(*B*) could have been calculated by storing the MCMC samples from the posterior distribution of the model parameters *μ*_*rs*_ and *β*_*rsk*_ produced by WinBUGS, and using other statistical software to calculate *E*(*C*) and *E*(*B*) in terms of these parameters. The WBDev approach saves the cost of storage and processing the stored samples, which may be expensive if there are large numbers of unknown parameters or if a large MCMC sample is required to represent the posterior distribution accurately.

## 4. Bayesian model assessment and model averaging

The alternative model structures that were listed in Section 2.4 may all fit the data reasonably well but still yield different expected costs and benefits. To enable this model uncertainty to be formally acknowledged in the decision about the most cost-effective intervention, we seek a measure of the adequacy *f*(**x**|*M*_*k*_) of each model *M*_*k*_, judged from the data **x**. We would like to use this measure to derive *model probabilities p*(*M*_*k*_|**x**) among the set of models being compared (Draper, 1995; Burnham and Anderson, 2002). These are used to calculate a *model-averaged* posterior distribution *π*{*z*(*θ*)|**x**}, for any function *z*(*θ*) of parameters *θ*, in terms of the model-specific posterior distributions *π*{*z*(*θ*)|*M*_*k*_,**x**}, which accounts for the uncertainty about the model choice (Draper, 1995). In this example, *z*(*θ*)=*E*(*C*) or *E*(*B*), and 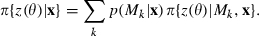
(4)

## 4.1. Predictive versus consistent model assessment

The usual Bayesian approach to model uncertainty (Draper, 1995; Kass and Raftery, 1995) involves computing *posterior model probabilities*. These are defined as proportional to a prior model probability *p*(*M*_*k*_) multiplied by the marginal likelihood, i.e. the likelihood for data **x** integrated over the prior distribution of parameters *θ*: 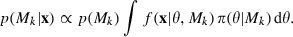
(5)

The prior and posterior model probabilities are interpreted as the probability of model *M*_*k*_ being true. As discussed by Bernardo and Smith (1994), judging model adequacy by posterior model probabilities is only suitable in an 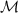 closed scenario, where it is believed that one of the candidate models is the truth, but it is not known which. Then this model selection procedure would be consistent—as the sample size increases for fixed priors, the model probabilities in expression (5) will converge towards a probability of 1 for the true model. Using a decision theoretic argument, Bernardo and Smith (1994), section 6.1.5, and Key *et al.* (1999) showed that model averaging using expression (5) gives optimal prediction or estimation when one of the models is assumed true.

But, in an 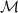
*open* scenario, candidate models are chosen to approximate a true model of unknown form. For example, the true model may be extremely complex. Then, more complex models would give better predictions in large samples, but these would be overpenalized by criteria such as expression (5) and its Bayesian information criterion approximation (Schwarz, 1978) which aim for consistency. To derive an optimal model choice criterion or estimation procedure in this situation, the true model must be approximated. This suggests model assessment using predictive principles such as cross-validation. Bernardo and Smith (1994), section 6.1.6, derived cross-validatory criteria for choosing a model to give optimal point predictions in an 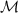 open scenario, although they did not discuss uncertainty about model choice.

In the health economic contexts that we consider, models are considered as convenient devices to approximate the highly complex processes of progression of disease and response to treatment. We assume that the true process underlying the data is too complex to identify completely even with an arbitrarily large sample. Therefore, instead of using expression (5), we take a predictive approach. We judge model adequacy by the expected utility *E*{*U*(**y**|**x**,*M*_*k*_)} of predicting a replicate data set **y** based on a model *M*_*k*_ fitted to data **x** (following Gelfand and Ghosh (1998)). In the following two sections, we describe two commonly used Bayesian model assessment measures of this type, based on different utilities. We then describe how sampling uncertainty about model selection using these measures may be accounted for, giving *model selection probabilities* which are used to compute a model-averaged posterior distribution.

## 4.2. Pseudo-marginal-likelihood

The utility function *U*(·) can be defined as the posterior predictive likelihood for **y**, i.e. the likelihood integrated over the posterior distribution of the model parameters *θ* (Laud and Ibrahim, 1995), 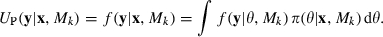
(6)

The expectation of this predictive utility for a replicate data set can be estimated, using only the sample data, by a cross-validatory predictive density (Geisser and Eddy, 1979), 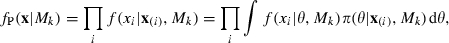 where **x**_(*i*)_ is all observations excluding *x*_*i*_. This is often termed the PML. It differs in aim from the marginal likelihood in expression (5), assessing predictive ability rather than fidelity to the data. In addition, it does not suffer the sensitivity to choices of the prior variance for parameters (‘Lindley's paradox’) that is experienced by the marginal likelihood (Gelfand and Dey, 1994).

Gelfand and Dey (1994) described an importance sampling method for estimating the PML based on a single MCMC model fit, which avoids the need to refit the model with each observation excluded in turn. (For ease of notation in this section, the dependence on the model *M*_*k*_ is omitted.) The full data posterior density *π*(*θ*|**x**) is used as a proposal distribution to approximate the leave-one-out posterior density *π*(*θ*|**x**_(*i*)_). Given an MCMC sample *θ*_1_,…,*θ*_*N*_ from the posterior of *θ*, the importance weights are then *w*_*ir*_=*π*(*θ*_*r*_|**x**_(*i*)_)/*π*(*θ*_*r*_|**x**)∝1/*f*(*x*_*i*_|*θ*_*r*_), and the importance sampling estimate of *f*(*x*_*i*_|**x**_(*i*)_) is the *harmonic mean* of *f*(*x*_*i*_|*θ*_*r*_) over the posterior sample: 
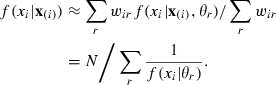
(7)

Thus, the quantity 1/*f*(*x*_*i*_|*θ*_*r*_) is monitored during MCMC sampling, and the estimate of *f*(*x*_*i*_|**x**_(*i*)_) is the reciprocal of its posterior mean. A sufficiently large MCMC sample should be taken to ensure that the posterior mean is stable.

## 4.3. Deviance information criterion

An alternative commonly used Bayesian model comparison measure is the *deviance information criterion* (DIC) (Spiegelhalter *et al.*, 2002). This is an estimate of an expected predictive utility *E*{*U*(**y**|**x**,*M*_*k*_)} of a similar form to equation (6), defined as 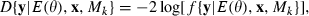 the predictive deviance based on ‘plugging in’ the expected parameter values of model *M*_*k*_. Since the utility *U*_D_(**y**|**x**,*M*_*k*_)=*f*{**y**|*E*(*θ*),**x**,*M*_*k*_} does not consider uncertainty about the parameter values, as *U*_P_ (6) does, the DIC is expected to be lower than the −2 log (PML) (reflecting lower loss, or higher estimated utility).

The deviance 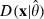 of the data **x** at the posterior mean 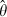 is an underestimate of *D*{**y**|*E*(*θ*),**x**,*M*_*k*_}, with asymptotic bias 2*p*_*D*_, giving 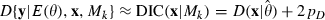 where 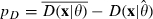 is the ‘effective number of parameters’ and *D*(**x**|*θ*) is the posterior mean deviance. The asymptotic argument assumes that the models under consideration are reasonable approximations to the true process, and that the posterior distribution of *θ* is asymptotically normal. Therefore a parameterization should be chosen so that *θ* has an approximately normal posterior. In our example, the posterior means of log (*p*_*ijrs*_/*p*_*ijr*1_) (equation (1)) were used to compute the plug-in deviance 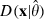. Plummer (2008) provided a more formal justification for the DIC, based on a cross-validation argument which is valid when the effective number of parameters is much smaller than the number of observations. This is true for our example, with about 60 parameters and over 4000 observations.

For models with weak prior information and approximately normal likelihoods, 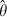 will be close to the maximum likelihood estimate and *p*_*D*_ will be close to the true number of parameters. Then the DIC will be approximately equivalent to Akaike's information criterion AIC, 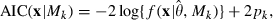
−2 times the maximized log-likelihood penalized by twice the number of parameters *p*_*k*_ in model *M*_*k*_ (Akaike, 1973).

## 4.4. Predictive model averaging

Model assessment by posterior model probabilities (5) naturally provides the weights in equation (4) for model averaging to account for uncertainty about model selection. But since we assume that the true model is too complex to be identifiable from an arbitrarily large sample, we prefer to compute a model-averaged posterior in which greater weights are given to models with better predictive ability (according to the PML or DIC) rather than models with higher posterior probabilities of being true.

It is not immediately clear how to account for model uncertainty when models are preferred according to their predictive ability. Laud and Ibrahim (1995) and Ibrahim *et al.* (2001) calculated the standard deviation of predictive model selection criteria to ‘calibrate’ differences in the criteria between models, but they did not discuss how to proceed when there is doubt about which model is best and the models give different inferences. By analogy with the marginal likelihood in expression (5), one approach would be to take model probabilities proportional to the PML *f*_P_(**x**|*M*_*k*_). It has also been suggested (Brooks, in the discussion on Spiegelhalter *et al.* (2002)) that model probabilities could be defined as proportional to a transformation of the DIC, 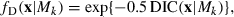 by analogy with the ‘Akaike weights’ exp {−0.5 AIC(**x**|*M*_*k*_)} that are often used for frequentist model averaging (Akaike, 1978; Buckland *et al.*, 1997; Burnham and Anderson, 2002). However, the performance of model-averaged estimators based on either *f*_P_(**x**|*M*_*k*_) or *f*_D_(**x**|*M*_*k*_) has not been studied, and it is not clear how the model probabilities should be interpreted. Burnham and Anderson (2002), chapter 6, suggested that model averaging using Akaike weights can be interpreted as a Bayesian model averaging procedure with an implicit prior *p*(*M*_*k*_) over the model space which favours larger models for greater sample sizes. Such a prior cannot be interpreted as a degree of belief in the truth of the model—rather it represents the belief that the model will give the best predictions on a replicate sample.

Instead, we use model averaging weights *p*(*M*_*k*_|**x**) which correspond to the probability that model *M*_*k*_ is selected by the predictive criterion, in other words, the probability that model *M*_*k*_ gives the *best predictions on a replicate data set, among the models being compared*. This probability reflects the sampling uncertainty arising from reusing the data to assess future predictive ability. This model selection probability can be estimated by a bootstrap procedure. The resulting model-averaged posterior (4) is a mixture of the posteriors that we would obtain if we repeated the study a large number of times with replicate data sets from the same process and based the results on the ‘best’ model at each repetition. Buckland *et al.* (1997) and Burnham and Anderson (2002) also estimated probabilities for model averaging in this way, as the proportion of bootstrap resamples in which each refitted model had the lowest AIC among the models. Breiman (1996) described a similar *bagging* (‘bootstrap aggregating’) procedure for improving predictions by combining models.

## 4.4.1. Bayesian bootstrap

To avoid the computational expense of refitting models to calculate the model selection probabilities, we use a Bayesian bootstrap method that was described by Vehtari and Lampinen (2002). Instead of sampling with replacement from **x**, the Bayesian bootstrap samples sets of probabilities *q*_*i*_ that the random variable *X* underlying the data takes the value of each sample point *x*_*i*_. In one bootstrap iteration, samples 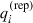 of *q*_*i*_ are drawn from a ‘flat’ Dirichlet distribution with all parameters 1. This is the posterior distribution of *the distribution of X*, conditionally on the sample **x** and an improper prior (Rubin, 1981). The bootstrap replicate of the sample statistic is then computed by using the original data **x** with weights of 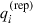.

In this example, the sample statistic is the log(PML), the sum of the log-predictive-ordinates for each point *x*_*i*_, 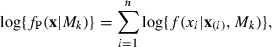 where *n* is the sample size. The Bayesian bootstrap replicate of the log (PML) is then 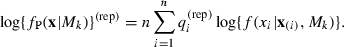
(8)

Similarly, the DIC can be decomposed into a sum over observations *i*, 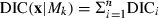, where 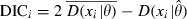, so the bootstrap replicate of the DIC is 
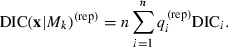


The advantage of the Bayesian bootstrap over a classical non-parametric bootstrap, for such statistics equivalent to sums over observations, is that there is no need to resample the data and to refit the model. The log {*f*(*x*_*i*_|**x**_(*i*)_,*M*_*k*_)} are calculated once by using equation (7), and at each bootstrap iteration they are summed with different weights by using equation (8). This procedure also assumes that any dependence between the *f*(*x*_*i*_|**x**_(*i*)_,*M*_*k*_) or DIC_*i*_, due to each one being a function of the data **x** via the posterior of *θ*, contributes a negligible amount towards the uncertainty surrounding the summed statistic.

Thus, a definition of *p*(*M*_*k*_|**x**) which corresponds to the uncertainty about model selection arises naturally from the bootstrap procedure, as the proportion of bootstrap samples for which *M*_*k*_ has the highest log(PML), or the lowest DIC, among the models considered. We label these as *p*(*M*_*k*_|**x**)^(PML)^ and *p*(*M*_*k*_|**x**)^(DIC)^ respectively. These probabilities have a natural interpretation as the probability that the model has the greatest expected predictive utility among the set of models being compared.

## 5. Results of the implantable cardioverter defibrillator study

The base case model *M*_1_ and the alternative models *M*_2_–*M*_10_ that were described in Section 2.4 were fitted to the ICD data. 25 000 MCMC iterations were used to calculate posterior summary statistics following a burn-in of 5000 iterations. The posterior means and 95% credible intervals for the incremental cost and QALY associated with ICD therapy are presented in Table 2, alongside the model adequacy measures −2 log (PML) and the DIC. Lower values of −2 log (PML) and DIC indicate better-fitting models. Note that the ‘effective number of parameters’*p*_*D*_ is less than the number of parameters given in Section 2.4, because of the constraints that are imposed by the prior distributions (Section 2.2).

**Table 2:** Lifetime cost-effectiveness analyses and model adequacy statistics for alternative models of the ICD data†

*Model*	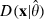	*p*_*D*_	*DIC*	−2*(PML)*	*p*(*M*_*k*_|**x**) *PML*	*p*(*M*_*k*_|**x**) *DIC*	*Incremental cost*Δ_C_*(£)*		*Incremental QALY*Δ_B_		*ICER per QALY*	*PCE (£ 20000)*
*M*_1_, base case	14604	57	14717	14742	0	0	31709	(28362,36073)	1.89	(0.37,3.43)	16763	0.67
*M*_2_, age effect on length of stay	14575	69	14712	14745	0.16	0.24	31488	(28001,36251)	1.90	(0.45,3.41)	16555	0.68
*M*_3_, fewer effects on amiodarone toxicity	14608	54	14716	14739	0.07	0.02	31527	(28282,35794)	1.89	(0.36,3.35)	16711	0.67
*M*_4_, fewer sex effects	14614	53	14719	14742	0.05	0.01	31600	(28236,35944)	1.92	(0.44,3.39)	16478	0.69
*M*_5_, quadratic age	14604	56	14716	14744	0.01	0.01	38889	(31209,49972)	1.32	(0.21,2.60)	29454	0.13
*M*_6_, cubic age	14591	62	14715	14740	0	0	39056	(30893,51743)	1.17	(0.11,2.34)	33384	0.07
*M*_7_, quartic age	14571	65	14701	14726	0.66	0.47	48921	(31256,151888)	1.29	(0.15,2.87)	37984	0.06
*M*_8_, quadratic age × treatment	14592	67	14726	14759	0	0	30919	(27314,36501)	1.29	(−0.80,3.84)	23936	0.36
*M*_9_, cubic age × treatment	14567	76	14720	14756	0	0.01	23064	(−54902,34631)	0.48	(−1.92,2.09)	48199	0.15
*M*_10_: quartic age × treatment	14537	85	14707	14749	0.04	0.24	13961	(−144025,34098)	0.20	(−2.76,1.87)	70520	0.17
Model average using *p*(*M*_*k*_|**x**)^(PML)^							42589	(28055,114736)	1.42	(0.02,3.16)	29987	0.23
Model average using *p*(*M*_*k*_|**x**)^(DIC)^							35754	(−2495,93057)	1.18	(−1.28,3.06)	30364	0.25

†Posterior means and 95% credible intervals are presented where appropriate. ICER is the incremental cost-effectiveness ratio, defined as the ratio of the posterior means of Δ_C_ and Δ_B_. PCE(*λ*) is the probability that ICD is cost effective if a decision maker is willing to pay at most *λ* (see equation (3)).

## 5.1. Adequacy of the competing models

**Fig. 3:**
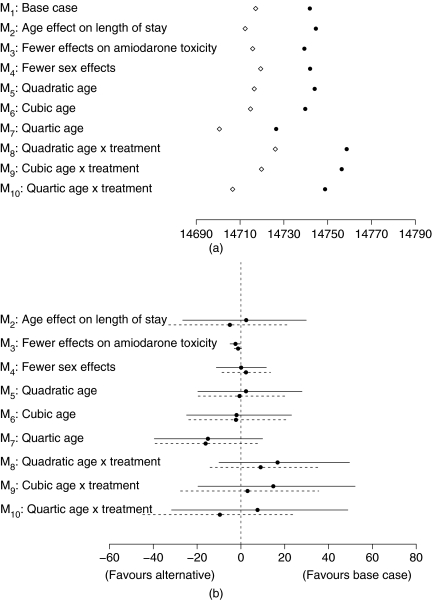
(a) Comparison of measures of adequacy, the DIC (⋄) and mean −2 log (PML) (•), for 10 competing models of the ICD data and (b) mean and 95% interval estimates for the difference in −2 log (PML) (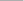) and the DIC (- - - - - -) of each alternative model from the base case *M*_1_

The DIC and −2 log (PML) are compared in Fig. 3(a) for models *M*_1_–*M*_10_. Owing to the different utilities which they estimate, the DIC values are lower than the −2 log (PML). The relative preferences for the various models, as expressed by −2 log (PML) and the DIC, are broadly similar, though the DIC does not penalize the extra complexity of *M*_8_–*M*_10_ as much as the −2 log (PML), perhaps because the utility underlying the DIC ignores parameter uncertainty, and these models are more weakly identifiable. The model with the best predictive utility, judged by both measures, is model *M*_7_, in which mortality is a quartic function of age. The extra complexity of models *M*_8_–*M*_10_, with interactions between age and treatment, does not lead to improvements in fit compared with *M*_5_–*M*_7_.

The bootstrap procedure (Section 4.4.1) is used to produce a set of 1000 replicates of both −2 log (PML) and the DIC for each model under sampling uncertainty. Since it is the difference in the criterion between models which is of interest for model comparison, rather than its absolute value, the uncertainty surrounding the criteria is not presented in Table 2. Instead, credible intervals for the difference in −2 log (PML) and the DIC of each alternative model from the base case are illustrated in Fig. 3(b). This suggests that, for the majority of bootstrap samples, model *M*_7_ has a lower −2 log (PML) and DIC than the base case, indicating better fit. Model *M*_8_, in contrast, has a generally worse fit.

The model selection probabilities *p*(*M*_*k*_|**x**) are presented in Table 2. The probability that each model has the highest predictive utility among the models being compared, calculated from the PML and DIC (Section 4.4), is given as *p*(*M*_*k*_|**x**)^(PML)^ and *p*(*M*_*k*_|**x**)^(DIC)^ respectively. The model *M*_7_, with mortality as a quartic function of age, is given the greatest weight by the PML-based method at 66%, followed by model *M*_2_ at 16%. Under DIC model selection, the probability of *M*_7_ is lower at 47% but still has the greatest weight, whereas model *M*_10_ has a greater probability of selection at 24% instead of 4%. These bootstrapped model probabilities are very different from the less interpretable probabilities that are derived by drawing analogies between PML or DIC and established model averaging methods (Section 4.4). Calculating *p*(*M*_*k*_|**x**) as proportional to the PML, by analogy with model averaging using marginal likelihood, gives a probability of over 99% to model *M*_7_. Calculating *p*(*M*_*k*_|**x**) as proportional to exp (−0.5 DIC) gives a probability of 95% for *M*_7_, and 5% for model *M*_10_. The sampling uncertainty surrounding the measures of fit is important in this example. The quartic model *M*_7_ was observed to give the best fit to the ICD data, but Fig. 2 suggests that this preference may have been strongly influenced by the small number of deaths beyond 80 years. Bootstrapping the estimate of expected fit to a replicate data set moderates the contribution of these outlying sample points to the model weight.

## 5.2. Inferences from the competing models

**Fig. 4:**
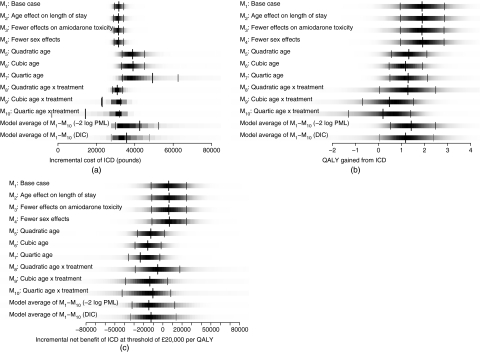
Posterior distributions of (a) incremental cost, (b) incremental QALY and (c) incremental net benefit for *λ*=£20000 of ICD compared with AAD therapy, under 10 alternative models and two model-averaged distributions: the intensity of black shading at each point is proportional to the posterior density, using a kernel density estimate, with each posterior mode shaded black (as in Jackson (2008)); longer ticks indicate the posterior mean and shorter ticks indicate the 10% and 90% quantiles (note that the mean incremental cost is just inside the lower quantile for the skewed cost distribution under *M*_9_)

The posterior distributions of the incremental cost *E*(*C*), incremental QALY *E*(*B*) and the incremental net (monetary) benefit, defined as *λ*(*B*)−*E*(*C*) for a ‘willingness-to-pay’ threshold of *λ*=£20 000 per QALY (which is conventionally used in the UK; see National Institute for Health and Clinical Excellence (2008)) are illustrated in Fig. 4, and summarized in Table 2. The incremental cost and incremental QALY arising from the base case *M*_1_ and the three alternative covariate selection scenarios *M*_2_–*M*_4_ are very similar—there is little uncertainty about the substantive conclusions arising from covariate choice. In contrast, extrapolating the age effect on mortality by using polynomial curves, instead of assuming constant risk after age 70 years, leads to smaller expected incremental QALYs for ICD therapy and increased incremental costs, and thus lower incremental net benefits and an increased ICER per QALY, from £17000 for the base case to £38000 for the quartic model. The probability PCE(*λ*) that ICDs are cost effective, if a provider is willing to pay *λ*=£20 000 per QALY, is reduced from 0.67 under the base case to 0.06 for the quartic model (Table 2). Including extra age–treatment interactions in the polynomial models leads to fairly similar incremental costs to the base case but lower estimates of QALYs gained, leading to higher estimated ICERs and lower incremental net benefits, but these models are less well supported.

## 5.3. Model-averaged analysis

The different inferences that were obtained from the competing models are combined into a model-averaged analysis, which weights the models according to their adequacy or predictive ability. Samples from the model-averaged posterior distributions of the quantities of interest are obtained by combining proportions, defined by *p*(*M*_*k*_|**x**)^(PML)^ or *p*(*M*_*k*_|**x**)^(DIC)^, of the MCMC samples from the posterior distributions from models *M*_1_–*M*_10_. These are illustrated alongside the model-specific posterior distributions in Fig. 4. Since 66% of the total contribution to the model-averaged posterior using PML weights comes from model *M*_7_, the posterior distributions are fairly similar to those from *M*_7_, but with a slightly lower cost, greater QALY gained and greater net benefit. The credible intervals are wide, reflecting the uncertainty about the model choice. The DIC-based model-averaged cost and QALY estimates are lower, because of the greater contribution from model *M*_10_, but the incremental cost-effectiveness ratios are very similar for the two model averages. The model-averaged probability that ICD therapy is cost effective for *λ*=£20 000 per QALY is 0.23 by using the PML weights and 0.25 by using DIC weights, compared with 0.06 for the single best-fitting model *M*_7_, and 0.67 for the base case (Table 2).

## 6. Discussion

Bayesian methods implemented with MCMC sampling are well suited to health economic decision modelling. Samples can be drawn directly from the posterior distribution of the expected cost and effectiveness, which is a complex function of the model parameters. This accounts formally for the uncertainty surrounding the model parameters. In this paper we have shown how this can be accomplished, even for complex models, within routinely used software for MCMC sampling. We have also shown how uncertainty about model structure can be accounted for in Bayesian cost-effectiveness models. Models can be compared by their expected predictive utility, estimated by the cross-validatory PML or the DIC, which can be computed routinely as part of MCMC sampling. The PML does not rely on asymptotic approximations, unlike the DIC. However, the DIC is in general easier to calculate than the PML, which relies on the convergence of the harmonic mean of the predictive ordinates (Section 4.2). The harmonic mean is unstable in some circumstances, though improvements have been proposed (Newton and Raftery, 1994; Kass and Raftery, 1995).

These measures can be used to construct a model-averaged posterior distribution which includes structural uncertainty. Model weights can be estimated by a bootstrap method, as the probability that each model is selected by the predictive criterion, rather than the standard Bayesian method which weights each model by its posterior probability of being true. We assume that the true model is extremely complex, so that posterior model probabilities would overpenalize complex models which would lead to better predictions in larger data sets. The model selection probabilities that we compute have an easier interpretation than weights calculated, by analogy with established model averaging methods, as proportional to the PML or exp (−0.5 DIC). Another advantage of a model averaging weight which allows for sampling uncertainty is to moderate the influence of outliers on the assessment of model fit, as in our example (Fig. 2).

Assessing long-term cost-effectiveness involves combining evidence from data subject to a range of complexities and uncertainties. In the ICD example presented, there are several such issues which have not yet been addressed, but for which the Bayesian formulation can be extended. In this discussion we briefly describe some of these possible elaborations. We also discuss alternative methods of accounting for model uncertainty.

## 6.1. Choice of states

A common structural uncertainty in cost-effectiveness decision models is the choice of clinical states which comprise the Markov model for cost and benefit accumulation. In the ICD analysis there are six different causes of hospital admission which are assumed to incur different costs. If the costs and utilities for two potential states are similar, then it may not be necessary to distinguish between those two states for estimating lifetime cost and effectiveness. Similarly, if the two treatment groups have similar incidences of a particular state, then that state will not affect the difference in cost and benefit between the treatments. Then it would not be necessary to include that state in the model at all. As a sensitivity analysis, two alternative state choices were considered for the ICD study.

- *M*_11_, the two hospital admission states corresponding to non-arrhythmic cardiac causes and non-cardiac causes are merged. The unit cost for the combined state is an average of the costs for the separate states, weighted by the incidence of each state during the study.
- *M*_12_, these two hospital admission states are removed from the model, so that a period in hospital for those causes is considered to be the same as the ‘out-of-hospital’ state.

Hospital admission for arrhythmia is retained as a distinct state, since the two therapies being assessed are intended to prevent arrhythmia. ICD maintenance or replacement and drug toxicity are expected to be important sources of extra cost for ICD and AAD therapy respectively.

The results of both of these models were not substantially different from the base case (posterior mean incremental cost £ 31619 for model *M*_11_, £ 31678 for *M*_12_, and incremental QALY gained 1.90 and 1.91 respectively). It is not clear how the fit of models on different state spaces should be compared. The criteria that we have described cannot be used to compare models *M*_11_ and *M*_12_ against *M*_1_–*M*_10_, since they were fitted to different data sets, with transition counts aggregated for the states merged. Likelihoods for different data sets are on different scales. An alternative approach would be to compare the ability of the models to predict observed quantities such as the short-term cost actually incurred or the quality-adjusted survival time, which have the same interpretation under each model. Expert judgement about whether an event has a distinct cost and utility from similar events, or whether treatment groups are expected to differ in their incidence of that event, could also be included.

## 6.2. Missing data

Missing data are encountered routinely in medical studies. 78 patients were omitted from our ICD analysis since their LVEF at baseline was missing. We fitted a straightforward extension to our model which included a Bayesian multiple imputation for this covariate. A regression model in terms of all other covariates was used to estimate a fixed prior probability of low LVEF for each individual with missing LVEF. This enabled a value to be imputed for these individuals at each MCMC iteration. Using the base case model, there is little difference in the estimated incremental QALY gained (posterior mean, 1.88 years; 95% credible interval, 0.47–3.31), but about a 10% difference in the estimated cost (posterior mean, £34598; credible interval, £30589–39795). Therefore there may have been a slight bias caused by omitting individuals with missing covariates.

In addition, 154 UK ICD patients were omitted from the data because their hospital admission histories were unknown. The remaining information from these patients is more difficult to include in the analysis. Since a greater proportion of patients with missing hospital data (28%) died compared with those with complete data (10%), ignoring missing hospitalizations may have biased the results. An imputation model for hospital admission histories could be constructed, but predicting from this model would result in a variable number of daily transitions from each state, i.e., although in each imputed data set an individual would have a fixed number of daily transitions from the start of treatment until their known death or censoring time, the denominators *N*_*ijr*_ of the multinomial model (1) from each source state *r* would vary between imputations. It is not clear how the fit of a model to such data could be assessed.

## 6.3. Extrapolation assumptions

The most important model uncertainty in our example is the dependence of mortality on age. Data on older ages are sparse, and different models extrapolate differently beyond those ages (Fig. 2). A polynomial of degree 4 provided the best fit to short-term data in this example, but the true age–mortality relationship is likely to be more complex. Population mortality statistics can often be incorporated in health economic studies to improve estimates of long-term survival (Demiris and Sharples, 2006). In Fig. 2, the log-odds of death within a day are plotted against age for the male and female populations of the UK (data for 2003–2005 from the UK Government Actuary's Department, Web site ), alongside the same data for the ICD study patients. The Canadian population survival data for 2000–2002 (which are not shown, but can be obtained from Statistics Canada, Web site ) were almost indistinguishable from the UK data. If the odds ratio between the population and study data were constant across ages, then this could be estimated and used to inform extrapolations of the survival probability for study patients, e.g. as a prior distribution. This odds ratio for all-cause mortality is clearly not constant in this example, but it is more likely that it would be constant for mortality from causes other than cardiac arrhythmia, or from non-cardiac causes. To make use of this information, the Markov model (Fig. 1) would have to be extended to include separate states for arrhythmic or cardiac mortality and other causes of death.

In health economic decision models which involve assessing long-term cost-effectiveness beyond the horizon of available data, the most important assumptions are generally the extrapolations of parameter values estimated from short-term data. Some of the uncertainty about these assumptions can be accounted for by model averaging based on assessments against short-term data. But assumptions about future changes in progression of disease, mortality and treatment effects are generally untestable. Therefore it may be illuminating to present a series of sensitivity analyses to demonstrate the effect on the results if key parameters were to change.

## 6.4. Other sources of uncertainty

Another major source of uncertainty in health economic models is the choice of data to use to inform a particular parameter such as a treatment effect. Ideally all relevant evidence should be used, but this may involve combining studies with slightly different populations, different interventions or outcomes from those of interest. As shown by Turner *et al.* (2009), expert judgements may be used to inform about the biases from each piece of evidence. These can be incorporated as prior distributions as part of a fully Bayesian random-effects meta-analysis.

Heterogeneity between individuals in their incidence of events or in the effects of treatment or other covariates may in principle be accounted for by extending the regression model (1) to a hierarchical model with exchangeable random intercepts *μ*_*irs*_ or random coefficients *β*_*irsk*_. Models could be assessed by using the DIC, which was motivated as a method for comparing hierarchical models where the effective number of parameters in the model is not clear, but alternatively the PML may also be computed by using the methods that were described in Section 4.2.

The expected cost and effectiveness of a policy implemented for a real population may also vary from that predicted for a fixed ‘typical’ patient described in Section 2.3. This extra variability may be estimated easily by replacing the fixed patient definition by a probability distribution before performing probabilistic sensitivity analysis.

## 6.5. Alternative methods for model uncertainty

An alternative to bootstrap-estimated probabilities of model selection would be to estimate model averaging weights by optimizing the utility for a model-averaged estimate or prediction. This would extend the decision theoretic methods that were described by Bernardo and Smith (1994) to determine an optimal estimate or prediction in an 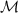 open scenario using a combination of all plausible models, instead of a single model choice. This is related to the ‘stacking’ method, which was described by Wolpert (1992) in the context of machine learning, which derives model-averaged predictions by choosing weights to minimize cross-validatory squared error.

An alternative to directly using predictive criteria for model assessment would be to use Bayesian model averaging methods based on marginal likelihood as in equation (5), but with a suitable prior *p*(*M*_*k*_) over the model space, as described by Burnham and Anderson (2002), chapter 6. The prior would acknowledge that, when the underlying data-generating process is complex, then, as the sample size increases, larger models will have better predictive ability. Such priors, and the resulting posterior model probabilities, have a pragmatic interpretation as the strength of belief in the expected predictive ability, rather than the truth, of a model. Reversible jump Markov chain Monte Carlo sampling (Green, 1995) is another technique for including model uncertainty which gives similar inferences to model averaging by using marginal likelihoods (Han and Carlin, 2001). This constructs a single Markov chain to sample from a posterior distribution over the joint model and parameter space, which avoids the computational expense of fitting each model under consideration separately.

In the ICD example, the choice between models *M*_1_–*M*_10_ is a choice between different linear predictors in equation (1), including polynomial functions of age. This may be considered as a variable selection problem. Another method of including model uncertainty for such problems would be to fit the maximal model with all possible predictors, but with a suitable prior on the coefficients *β* which would minimize the predictive variance of the fitted model, and could also incorporate substantive beliefs (Greenland, 1993). However, to our knowledge, the routine choice of such priors in the absence of substantive information has only been investigated in detail for normal linear models (e.g. Zellner (1986), George and McCulloch (1993) and George and Foster (2000)).

Any consideration of model uncertainty involves choosing a reasonable set of models to compare or average over. As discussed by Draper (1995), a set of well-supported models which give different inferences should be chosen. In the ICD example, because of the computational expense of fitting one model, only three alternative covariate selections (Section 2.4.1) were chosen by judgement, and three alternative models for the dependence on age were chosen to encompass a range of different extrapolations (Fig. 2). But other models may have fitted better and led to different conclusions. Efficient automatic methods, such as reversible jump MCMC sampling, would potentially be useful for exploring larger model spaces.
